# Developing a Partnership Model to Address Gaps in Rural Healthcare Provider Training Using Simulation-Based Health Professions Education

**DOI:** 10.7759/cureus.36789

**Published:** 2023-03-28

**Authors:** Samyah Siraj, Ginny Brunton, Artur Arutiunian, Gordon Brock, Adam Dubrowski

**Affiliations:** 1 Health Sciences, Ontario Tech University, Oshawa, CAN; 2 Family Practice, Centre De Sante Temiscaming, Témiscaming, CAN

**Keywords:** simulator, healthcare provider, healthcare provider training, simulation-based education, rural and remote, partnership model, three-dimensional (3d) printing, healthcare simulation, simulation in medical education

## Abstract

Rural and remote (R&R) healthcare providers experience difficulties accessing continuing medical education, including simulation, to improve their clinical competencies to address the diverse health needs of the rural Canadian population. At the same time, the College of Family Physicians of Canada (CFPC) has identified a need to shift toward a competency-based curriculum to increase access to clinical training using innovative, flexible methods, such as simulation. Simulation is a strategy that can be applied to facilitate this learning by allowing learners to practice clinical skills on a simulator. However, the high cost of simulators is not a practical solution to address the training needs of R&R healthcare providers. In accordance with one of the CFPC’s policy considerations, establishing partnerships between relevant sectors such as university research and innovation centers, for-profit organizations (FPO), and not-for-profit organizations (NPOs) to develop and distribute simulators to R&R healthcare providers can help reduce costs and address gaps in health professions education. Modern, Industry 4.0-related technologies such as three-dimensional (3D) printing allow for sustainable and affordable manufacturing of simulators, however, the tools and “know-how” to develop these simulators are currently limited mainly to university research and innovation centers in urban areas. To date, no simulation-focused partnership model exists that addresses how Industry 4.0 augmented simulation technology can make its way from university research and innovation centers into R&R healthcare settings. The proposed solution is to create a simulation-focused partnership model between university research and innovation centers, FPOs, and NPOs to improve the diffusion of Industry 4.0 augmented simulation technology to the R&R Canadian healthcare sector. Diffusing simulators from a research lab to R&R healthcare providers is a sustainable approach aligned with CFPC’s policy considerations to strengthen rural medical education, subsequently strengthening rural medical practice.

## Editorial

Background

Healthcare providers practicing in rural and remote (R&R) areas of Canada experience challenges in providing quality care that addresses the diverse healthcare needs of its rural population, including the technical skills needed for high-acuity low-occurrence (HALO) procedures, which are clinical procedures that are rarely performed yet, when needed, need to be done urgently [1]. One factor that impacts the level of care provided is the suboptimal access to state-of-the-art education in R&R settings. It is considered a vital societal need to educate healthcare providers to prepare them for R&R practice. Barriers to accessing state-of-the-art continuing medical education (CME) for R&R area physicians include the cost of both travel time and time away from medical practices during current times of shortages, in addition to the financial burden of traveling to often distant academic centers to access CME [1].

A reform to health professions education (HPE) is necessary to respond to challenges in the delivery of healthcare services as the level of training and competencies of healthcare providers correlates with them being able to adequately serve the population’s health needs [1]. A shift towards a competency-based curriculum in HPE is suggested to ensure learners are able to acquire experience in diverse learning and work environments to aid them in responding to the healthcare needs of the communities they serve. This will allow healthcare providers to give more comprehensive care and have more advanced procedural skills to reflect their broad scope of practice and a broad range of clinical procedures [1]. The College of Family Physicians of Canada (CFPC) highlights that specialized resources are limited in R&R areas of Canada and recognizes that providers need to focus on developing contextual competencies through relevant learning opportunities to practice in R&R clinical settings [1]. Using innovative, flexible methods to educate healthcare providers and prepare them for practice in R&R communities can create a stable health workforce [1]. One of the objectives to improve rural education programs identified by the CFPC is to develop tools that can help increase access to clinical training, with one such tool being simulation technology [1].

Simulation-based health professions education (SBHPE) is the use of a simulative aid to replicate clinical scenarios for educational purposes and is an adjuvant to clinical training [2]. SBHPE is a training strategy that has the capacity to significantly benefit healthcare providers by addressing healthcare needs in a practically and clinically relevant manner with immediate application in clinical practice [2]. A simulator is a device that allows the user to reproduce a phenomenon under test conditions that is likely to occur in real-world situations [2]. Simulators in the context of HPE allow a learner to practice specific technical “procedural” skills, especially skills that cannot be easily practiced on patients, such as suturing and inserting chest tubes, through multiple repetitions in a controlled environment, which can be synchronous or asynchronous. The learner can receive feedback from experienced mentors to improve and correctly advance their competency in a skill without endangering a patient [2]. Realistic and commercial simulators are often prohibitively expensive [2]. Although these simulators can be incorporated into primary healthcare settings to improve learners’ competence and increase patient safety, the cost of such technology is not practical for all rural hospitals and not-for-profit organizations (NPOs) to sustain. A more affordable and sustainable solution, such as 3D-printed simulators, is needed to integrate SBHPE into rural education to support healthcare provider training.

One of the policy considerations suggested by the CFPC is to apply a pan-Canadian approach by creating opportunities for policymakers, physicians, rural communities, academia, and other healthcare providers to collaborate and support the development of rural education and practice [1]. Establishing partnerships between university research and innovation centers, for-profit organizations (FPO), and NPOs to develop and distribute simulators to R&R healthcare providers can help reduce costs and address gaps in HPE [3]. Integrating these stakeholders in a sustainable collaborative process through developing a sustainable business model that focuses on meeting consumers’ needs can have positive social and economic impacts [3].

Current gap

Industry 4.0 is considered the fourth revolution in manufacturing through its use of emerging technologies that deeply integrate business and engineering processes to make production flexible, efficient, and sustainable in a way that maintains high quality at a low cost [3]. The emergence of Industry 4.0 allows for the manufacturing of affordable and sustainable three-dimensional (3D) printed simulators through a flexible and efficient production process [3]. Simulators developed using 3D-printed and silicone technology have the capacity to be adapted to accommodate the needs of the provider based on the clinical skill they intend to practice [2]. The method of customization using additive manufacturing techniques allows for a reduction in the cost of producing the simulator [3]. However, Industry 4.0 tools to develop simulators are currently limited mainly to university or hospital-based research and innovation centers in urban areas, which if diffused to R&R settings can create a more significant impact.

To date, there are no specific partnership models with a focus on SBHPE that address how Industry 4.0 augmented simulation technology can make its way from university research and innovation centers into R&R settings. Existing models in the current literature have several gaps in addressing how simulators can be diffused from resource-rich university research and innovation centers to NPOs and hospitals in R&R areas. Specifically, the models lack a focus on SBHPE and simulation technology as a central element of the partnership as they primarily deal with pedagogy. Those with a simulation training component do not address the process of manufacturing the simulators that would be produced and delivered to R&R healthcare providers. In addition, limited literature can be found that discusses the funding stream that covers the cost of producing the simulators, how funding can be acquired, or how financial responsibilities are distributed amongst partners. An example of a partnership model that comes close to addressing the identified gap is the twinning partnership model, which is used to guide the collaboration between a university situated in a low-income country and a university in a high-income country [4]. Building a twinning partnership follows a six-stage approach: (1) build a partnership; (2) develop a work plan; (3) implement the program; (4) monitor outcomes (process, impact); (5) evaluate the results; (6) and disseminate the information [4]. This model outlines an effective process to implement collaborative programs between academic institutions and local NPOs through effective relationship building among partners and by creating a sustainable approach to meet the partners’ needs. This model is seen as an effective strategy for partnerships between institutions that focus on local stakeholders to guide the partnership, as it emphasizes building long-term relationships where partners equally contribute to find mutually beneficial solutions [4]. The limitations of this model concerning the identified gap are that this model does not have a specific SBHPE or technology component as part of the partnership and therefore provides limited information on the manufacturing process of the simulators. More specifically, the twinning partnership model does not guide on issues fundamental to technology development, funding, and intellectual property management that are fundamental to the diffusion of simulation technologies from urban to R&R settings.

Proposed solution

Public health systems research (PHSR) is a growing field of research in Canada that aims to examine the financing, delivery, and impact of public health services [5]. PHSR follows the philosophy of an integrated knowledge translation (IKT) approach whereby both researchers and decision-makers are engaged throughout the entire research process in a collaborative approach to produce relevant research findings [5]. One of Ontario’s top six PHSR priorities is partnerships and linkages, which emphasizes the importance of creating and mobilizing partnerships between various sectors (i.e., healthcare providers, educational institutions, community-based organizations, government, etc.) to improve the performance of the public health system [5]. This can be achieved by focusing research on understanding techniques to build partnerships across different sectors and evaluating the partnership outcomes, as achieved by the twinning partnership model. Prioritizing the establishment of multi-institutional partnerships between university research and innovation centers, FPOs, and NPOs can improve the capacity building of healthcare providers and knowledge exchange within the public health system [5]. Multi-institutional partnerships are an innovative approach to gather and optimize the use of resources from multiple institutions to provide quality educational opportunities to R&R healthcare providers [1]. To improve rural HPE and address the gaps in existing partnership models, the proposed solution is to develop an SBHPE-focused partnership model between university research and innovation centers, FPOs, and NPOs to improve the diffusion of Industry 4.0 augmented simulation technology to the R&R Canadian healthcare sector.

Developing a partnership model in the context of creating and delivering simulators to R&R healthcare organizations creates a win-win-win situation for each stakeholder involved. NPOs and hospitals in R&R communities benefit from building partnerships to increase accessibility to feasible simulators to provide adequate and continuing training to healthcare providers. The benefit to academic and research institutions is the opportunity to conduct interdisciplinary research. Research that is truly interdisciplinary and that is well suited for simulation in the R&R context is one that englobes individuals from different fields, who contribute ideas while, in the process, learning from and about each other. In doing so, they integrate their knowledge and adapt their methods from different disciplines, creating a synergy of knowledge and a synthesis of methodological approaches. Therefore, they are not working in silos and are trying to push the boundaries of methods, techniques, etc., beyond their home disciplines while discovering new pathways to mobilize technology [6]. FPOs benefit from this process by allowing a shift towards a social enterprise model. A social enterprise is a business with specific social objectives that serve its primary purpose. Such businesses seek to maximize profits while concurrently maximizing benefits to society, and the profits are principally used to fund social programs. Becoming a social enterprise benefits FPOs due to the taxation benefits whereby the FPO can shield itself from some taxes through their societal contributions. In addition, FPOs are able to tap into the academic sector, which provides them access to limited research and development (R&D) funding opportunities that can reduce or completely cover R&D costs. FPOs can also gain new business pathways by testing new markets inexpensively by engaging in low-risk, partner-driven, and low-cost partnerships.

Establishing a partnership between relevant organizations can facilitate the process of delivering simulators that are developed in a research lab to hospitals and educational institutions in communities that lack the resources. Developing simulators in a research laboratory using 3D printing technologies is an economical solution that helps reduce manufacturing costs and provides a sustainable SBHPE training approach [3].

Conclusion

The use of Industry 4.0 augmented simulation technology creates a path to develop feasible solutions to train healthcare providers in support of delivering the highest achievable standard of care in R&R parts of Canada. Producing low-cost, sustainable simulators addresses HPE gaps for R&R healthcare providers by providing them with the opportunity to increase their proficiency in clinical skills, in turn, strengthening rural medical practice. Developing a model to establish a partnership between relevant organizations to improve the diffusion of simulators will strengthen rural medical education and improve capacity building in the healthcare system.
